# Semantic physical science

**DOI:** 10.1186/1758-2946-4-14

**Published:** 2012-08-03

**Authors:** Peter Murray-Rust, Henry S Rzepa

**Affiliations:** 1 Unilever Centre for Molecular Science Informatics, Department of Chemistry, Lensfield Road, Cambridge, CB2 1EW, UK; 2 Department of Chemistry, Imperial College London, Exhibition Road Campus, London, SW7 2AZ, UK

## Abstract

The articles in this special issue arise from a workshop and symposium held in January 2012 (Semantic Physical Science’). We invited people who shared our vision for the potential of the web to support chemical and related subjects. Other than the initial invitations, we have not exercised any control over the content of the contributed articles.

## Background

The articles in this special issue represent an initiative to coordinate and develop the generation and use of semantic information in physical sciences, particularly chemistry, materials and earth sciences. Unlike bioscience, where semantic information and infrastructure is common, there has been relatively little effort and practice in physical science. A feature of physical science is its use of numeric quantities, coupled with fundamental quantities and systems of units. Although some designs (*e.g.* Dumontier [1], Adams/ChemAxiom [2], ThermoML [3]) have described ontologies for chemistry, and although we have created and deployed CML, there is relatively little other practice. The award of a “Pathways to Impact” grant from the EPSRC gave PMR the opportunity to run a workshop in this area and to explore the value and future of semantics. As we did with “Visions of a Semantic Molecular Future” [4] (VSMF) last year, we have agreed with BioMed Central that the proceedings of this workshop and the more general commentaries on semantics in physical science can be published as a thematic issue in this journal.

Much physical science is built on the systematic creation and collection of data. In this article we concentrate on “long-tail science” where standard methods are applied in many different laboratories. Although this accounts for large amounts of published work, the data are often not reported systematically and the experiments are not therefore formally reproducible. In many cases the data are collected to support a wider experimental program and can often be thought of as “analytical”.

It might have been expected that, with the advent of electronic data and widespread automation, the physical scientific community would have developed semantic interoperability. This has not happened, and the workshop concentrated on those areas which create large amounts of well-defined, modular output and where we believe semantics will add real value to the process. It also explores the semantic automation of running experiments and simulations (for example, in “parameter sweeps”) where a significant amount of material is represented by small variations in a common experimental program.

Traditional and current use of numeric data in physical science is often fragile as it is not expressed semantically. The human has to add their own layer of interpretation, often based on previous experience of recognising patterns. Anecdotally we repeatedly encounter cases where information components of an experiment are muddled or missing, or where it is it easy to input incorrect control and data information to a simulation program. By adding semantics we believe that there will be a very significant rise in the quality of the management, transport, interchange and storage of data in physical science.

In this our two efforts are instrumental output and calculation. In both of these, the practice has been commoditised so that it is possible for scientists to collect experimental data or calculate and simulate materials. Indeed relatively inexperienced scientists can achieve a high throughput of high quality data, although this can then raise the problem of misapplication.

### Promoting good practice

An outstanding example of what is possible has been demonstrated by the International Union of Crystallography (IUCr) over three or more decades. The IUCr represents and coordinates a specialist group of scientists who are involved in both fundamental new research and the provision of established methods as a validating technique for rapidly determining the structure of matter. They have coordinated efforts to standardise experimental techniques, data processing and structure solution. They have also given a great lead in the publication of data-rich science. They have outlined and in large parts implemented a workflow for the collection, validation and publication of a significant area in physical science.

Perhaps surprisingly, the rest of long-tail physical science has not implemented similar practices in data collection and computation. It is common for results to be mentioned in scientific papers but for the raw and the processed data to be unavailable. In some cases, the results are transformed for human view (*e.g.* molecular displays or graphical spectra) rather than being available in semantic form.

The primary focus of the workshop was to tackle a small number of areas where large amounts of highly valuable data are produced but not systematically collected, distributed and preserved or curated. Although the concept of re-use of semantic data is common in crystallography and biosciences, it is much less common in analytical chemistry and computation.

We invited a number of authorities who have been working in this area to explore the vision of more widely available semantic data and what we needed to do to make this happen. In particular, we concentrate on the development of dictionaries in a communal manner and have examined how this translates to other disciplines.

### The focus was therefore threefold

1. **Crystallography.** The IUCr’s CIF framework [5] serves as an excellent model for the rest of the discipline.

2. **NMR spectroscopy.** We estimate that many hundreds of millions of spectra are run annually but very few are Openly available in semantic form. There are databases such as NMRShiftDB [6] and BioMagResBank [7] which have created valuable resources but they are a very small proportion of the publishable data. The instrumentation is highly standardised and is either run as a service to chemists and other scientists or directly made available for them to carry out measurements. Although the practice of the science continues to develop, the fundamental physics is well understood and formally describable. We have chosen NMR not only because of its universality but its tractability (at least for the standard forms which account for 80% of the acquired spectra). If we are successful in this, the general semantic methodology should be relatively easily transferable to other types of spectroscopy and possibly chromatographic data.

3. **Computation and simulation.** This is now a very widespread technique for predicting the behaviour of unknown systems and for interpretation of experimental observations. It is increasingly possible for relative newcomers to run calculations and the rapid exponential growth of resources means that a very large number of systems in chemistry and materials science are now computable at acceptable cost. Computational chemistry represents a significant output of most of the large centralised supercomputing facilities. We shall address both gas phase calculations and condensed phases including regular crystalline solids.

### The articles in this thematic issue

The “papers” in this thematic issue explore the changing nature of scientific publication. They are not static, in that the authors have created a dynamically changing vision of their ideas and experience. We asked most of the contributors to provide a medium-length presentation (between 10–30 minutes) and we recorded all of these on video. All videos (listed below) have now been uploaded to one or more sites (University of Cambridge Streaming Media Service [8], DSpace@Cam [9], Vimeo [10] which provide a variety of outlets) under a CC-BY license to allow re-use. We have not transcribed them, but PMR has annotated them in blog posts so that different sections in them can be easily located. In addition, PMR has added his own comments in the blog posts so that readers can link through to ideas expressed here and in his commentaries.

• Introduction - Peter Murray-Rust [11]

• Why we (PNNL) are supporting semantic science - Bill Shelton

• Adventures in Semantic Materials Informatics - Nico Adams

• Semantic Crystallographic Publishing - Brian McMahon [12]

• Service-oriented science: why good code matters and why a fundamental change in thinking is required - Cameron Neylon [13]

• On the use of CML in computational materials research - Martin Dove [14]

• FoX, CML and semantic tools for atomistic simulation - Andrew Walker [15]

• Semantic Physical Science: the CML roadmap - Marcus Hanwell [16]

• CMLisattion of NWChem and development strategy for FoXification and dictionaries - Bert de Jong

• NMR working group - Nancy Washton

The videos record the participants’ thoughts before the workshop and, in some cases, modified by two days of workshop discussions (not all workshop discussions were summarised in presentations). Where appropriate, we have asked the authors to include links to the videos in their articles, but there is not a 1:1 correspondence between videos and articles.

This represents new opportunities in scientific communication and scholarly publications. Many of us would indeed regard these annotated videos as, at least in part, equivalent in impact to traditional “paper”-like discourse. They often contain a greater detail than a paper (for example, detailed slides), or, as in the case of Brian McMahon’s presentation, there are semantic interactive demonstrations (“cows”) which cannot possibly be represented in PDF (“hamburger”).

The symposium not only included the presentations but also ended in a very significant communal discussion on the value of semantics and how we could take it forward in physical science. At the end of this discussion we asked for an indication as to who would wish to contribute scholarly articles to a journal, and, as a result, have created this thematic issue. Some participants at the symposium did not present invited talks but offered to provide articles on semantic practice and design, and we are grateful to them.

The thematic issue, therefore, consists of articles some of which were partially pre-prepared for the symposium but heavily influenced by the two days of workshop proceedings. In addition, some of the articles arise directly out of the workshop and the symposium discussion. Many early versions of articles have been available to the group and have helped us to provide a balanced collection of mutually informed content. In some cases the process of writing the article has made major contributions to our understanding and practice. For that reason some are snapshots of work in continual progress.

In this overview, we’ve divided the articles into four general categories.

### Vision and visionary practice

Brian McMahon (article “Applied and Implied Semantics in Crystallographic Publishing”) provides the history and the formal basis of the CIF program. This acts as a vision for what is achievable in many areas of physical science.

Selected quotes from PMR’s blog post [17]:

“*I owe a huge debt to the International Union of Crystallography (IUCr) and Brian McMahon. Quite simply they are the best semantic scientific publishers of the current century. They also have the best community-base for scientific publishing that I know. The Union exists for its members and not for itself; its processes are as democratic as a scholarly body allows, and it is passionate about doing science properly.*

The IUCr has always had a major emphasis on data and terminology. It has run experiments on how reproducible crystallographic experiments can be. It spends much time on the basis of the science and how to describe it. For over three decades it has had initiatives in defining data representation. It’s blessed with the fact that modern instruments are highly reproducible and that crystallization is a classic method of purification. Because of that a crystal structure done in labs A and B is likely to be in very close agreement. There are exceptions – biological macromolecules are more heterogeneous – but generally it’s a highly reproducible science.

This tradition is now central to its publication ethos. Essentially every published result must be replicable (potentially falsifiable) from the information in the publication. Even 45 years ago (when I started) we were expected to type our raw data (thousands of observations) into the pages of the journals. Now it’s electronic but the bar has risen – we now have to publish the X-ray images. There is no room for subjectivity – and if the methodology is flawed the community WILL find it out.

The Union is committed to making crystallography accessible to everyone. For this reason it has advocated for 30 years that ALL publications (not just Acta Cryst.) should publish their crystallographic data. It’s moving towards Open Access and has a completely Open Access journal, Acta Cryst. E. In this journal the complete crystallographic experiment is checked, and if it’s apparently flawed it’s returned to the authors for comment. Every atom, every bond is checked.

[The APC for Acta Cryst E is $150]

*The reason that IUCr can do this, and why it is so highly regarded in all disciplines is that over the years they have steadily invested in the information infrastructure (ontology) of their discipline. And it’s been a community effort. Many people (Sid Hall, Howard Flack, Herb Bernstein, John Westbrook, and 30 others, including me)*[18]*have contributed in mails, meetings, software, specifications and lots more). Progress has been steady.*

And all of this has been designed, guided, glued together by Brian. And he’s done more – in the small Chester office of IUCr he and a few others have built a remarkable suite of publishing software. Fit for purpose, respected by the community of authors and readers/users alike. What other science can say that? (A very few, and I hope they’ll identify themselves here).

*And for me, IUCr/CIF/Brian have been a guiding light in the development of CML.*”

Unlike crystallography, compchem does not have a history of semantic publishing, and in his article “Chemical datuments as scientific enablers” HSR demonstrates how powerful the new technologies can be compared with traditional ink-on-paper. Henry has for many years pushed the boundaries of publication by creating semantic objects and supplying them as part of his published science. In last year’s VSMF paper [19], the BMC publishing process was initially unable to incorporate the semantic constructs that had been submitted, although it was all implemented some months after the conventional form of the article appeared on the journal pages. This follow up pushes the boundary further, and we are confident that the publisher will rise to the challenge.

### Semantically-enhanced Science

Martin Dove and colleagues (articles “Using CML in data analysis” and “Use of CML in scientific codes”) have been using CML for the last 10 years to support the computation of properties and behaviour of crystalline materials. He shows how CML makes more things possible in a shorter time, and thereby changes the nature of the science that he can do.

Markus Kraft and Weerapong Phadungsukanan (article “The Semantics of Chemical MarkupLanguage (CML) for Computational Chemistry: CompChem”) have worked with us for 4 years on supporting their calculations of combustion and other high-temperature processes through atomistic simulations.

Simon Coles (articles “Quantities, Units and Symbols in Physical Chemistry: The digital semantification of the Green Book“ and ”First steps in semantic descriptions of Electronic Laboratory Notebook (ELN) records”) runs the national crystallographic service at Southampton and has been using JUMBO for several years to semantify the output of the service. He has also been heavily involved in developing semantics for chemistry in the joint oreChem project [20], where we developed a semantic workflow.

### Implementation of semantics

A decade ago, we developed a formal basis for the semantics of physical science (STMML) [21] where we separated the infrastructure into:

• Basic ST(E)M data (datatypes and structure). This has proved resilient over the 15 years of deployment.

• Dictionaries. These drew very heavily from the IUCr practice.

• Conventions. This was envisaged in the early design but has only been seriously implemented in the last 5 years.

The STMML vision has proved to be adequate and valuable for large areas of physical science, and deserves to be in much wider use. For that reason, the articles give an insight into what is needed to implement it in areas beyond the primary disciplines in this issue.

Our own software implementation is sufficiently modular that other disciplines can use these components without having to include anything specifically chemical. This is reinforced by FoX’s support for keyhole markup language (KML) which is widely-used for geographical information (geo-tagging) systems (GIS) [22].

We have always appreciated that it was necessary to provide an implementation framework before being able to persuade significant numbers of the community that this was an effective way to go. We are now in the position where there are many systems which can read and/or write CML, and a significant number of publicly available libraries to support this process. In the article “Building a CML code library” we examine the features of current systems. There is no “best way” to write a CML code library, requiring as it does a balance between comprehensiveness, ease of implementation, ease of use and formal compliance, and we review this in depth in the article. We note that, as creators of CML, we have a responsibility to define the language, semantics and conformance, and the schema and software in the CMLXOM/JUMBO implementation act as the reference model.

A critical factor in the adoption of CML by the computational materials community was the ability to interface Fortran-based programs into a semantic framework. This was started some years ago, firstly by Alberto García and Jon Wakelin and subsequently by Toby White in the Materials Grid project [23]. Andrew Walker took over from Toby in 2008 and now has a critical mass of users and ongoing momentum. There is a significant section on FoX in the “Building a CML code library” article and the workshop discussed the most important new areas for FoX to address. At least ten programs have been fully or partially converted to use FoX and the workshop was able to make rapid progress in adding NWChem to the list (article “CML Semantics in Computation Chemistry: NWChem”). Bert de Jong is leader of the NWChem computational code project [24], one of the few that is available under an OS license. He is committed to CMLifying the program and to coordinating the development of dictionaries in semantic computation.

Because most of the current practice involves legacy (non-semantic) documents, it is necessary to provide convenient methods for converting these into CML. Over the last decade we have developed a framework of converters and other processing software (JUMBO-Converters) for the semantification of legacy data, including large and complex documents (article “JUMBO-Converters: a suite of parsers for chemical and other scientific data”).

The Chem4Word project [25] (article “Chem4Word”) represented the greatest in-depth and most modern approach to CML semantics. It included the requirement for all quantities to be formally linked to dictionaries and for all CML documents to be valid against conventions. The CMLLite convention [26] was developed for this purpose and serves as the archetype against which the convention specification and validation [27] (*e.g.* through XSLT stylesheets) were created. Chem4Word also used a stateless model based entirely on representing the state of the system at any stage by a CML document; there was no other data model in the system.

CML evolves in response to significant demands from the community. In most cases this can be done by using the current primitives of CML and creating appropriate dictionaries and conventions.

Marcus Hanwell and Geoff Hutchison (article “Avogadro: A Semantic Framework and Cross Platform GUI for Building Molecular Structures and the Analysis of Output”) are the primary authors of the Avogadro software [28] for displaying, building and computing molecular and crystal structures. They have adapted Avogadro to the semantic structure of CML so that it not only extracts the molecular structure but also displays its semantic environment and history.

The Quixote system figured prominently in the workshop but was described in the last thematic issue [29]. Since then, we have published [30] the formal basis of the Chempound [31] repository (the engine for Quixote), which allows the storage of arbitrarily large and complex semantic objects such as the output of computer programs [32] and crystallographic experiments [33]. By converting the data into RDF, it’s possible to provide a powerful Semantic-Web-friendly indexing system. Chempound has been designed to validate data files on ingestion so that both authors and consumers have very strong confidence in what is held in the repository.

### The Future

Tim Berners-Lee’s vision of the Semantic Web is built on several layers of design and technology and ultimately involves ontological systems that can apply formal logic to reason over data. We made a deliberate design decision to implement ontologies through the CML dictionary system, which does not have this semantic power but is capable of being extended to more powerful ontologically-based systems. In fact physical science, unlike bioscience, has a very well-established implicit ontological framework (*e.g.* scientific units).

Bill Shelton runs an analytical and computational group at Pacific Northwest National Laboratory (PNNL) [34] where he is committed to making data widely available. The article (with Nico Adams from CSIRO) “Semantic Physical Science - Making Physical Science Data Accessible and Useful” shows why semantics are essential for this activity.

The eScience Centre at STFC Rutherford Appleton Laboratory are responsible for developing the semantic infrastructure to support the latest high-throughput experiments and simulations carried out at a national laboratory. They have christened their approach Semantic Data Processing (SDP), and describe its benefits, impact and future challenges for large scale science in the article “Lightweight Semantic Data Processing for Facility Science”.

### MathCML

As often happens, the discipline of writing papers formally generates significant new ideas and insights. In the current case, as we were writing the semantic forcefield paper, it became clear that there was a major representational advance possible by combining MathML and CML. MathML is capable of expressing the mathematical foundation of all of the physical science covered in this issue. For example, the GULP user manual [35] deals with a very large number of different approaches to forcefields and gives their functional forms, and often parameterisation, in detail. It is now possible to encode large parts of such a manual into completely semantic declarative markup. The use of freely-available semantic math editors such as Formulator Mathml Weaver [36] means that a scientist can represent the mathematical concept in semantic form with no more effort than using a normal equation editor.

We have addressed this by writing a companion tool to JUMBO, JUMBO-MathML, which builds a semantic, sub-classed DOM using the subset of content-MathML vocabulary most relevant to physical science. It includes basic types and operations such as algebra, trigonometry, coordinate geometry, sets, differentiation and integration, and through its markup we can link to appropriate dictionaries. In correspondence with the MathML community, we agree that there is no single semantic approach for binding MathML into declarative programs, and MathCML takes a pragmatic approach with a certain amount of implicit semantics which will be natural to the physical scientist.

The semantic maths can be extended to manage sets of molecular information such as molecules and bonds. Since MathML is largely symbol-based, we can map the chemistry onto the lexical form whilst still creating valid MathML. MathCML contains prototypes for normalisation, validation and differentiation of mathematical expressions. This allows us, for example, to evaluate on-the-fly, the combination of a molecule, its atom typing, its geometry with the semantic forcefield and functional form, giving rise to a molecular energy calculator. The forcefield can be differentiated, so that we can use analytical derivatives for optimising the energy minimisation and for calculating forces for dynamics trajectories. The coding of derivatives, especially second and third, has traditionally required an enormous amount of human effort, and has been a deterrent to scientists who wish to introduce new functional forms but do not have the resources to create several thousand lines of analytically-correct code.

In contrast, MathCML, combined with the declarative description of a problem, allows any scientist to define their problem and evaluate basic properties. Our early prototype (the “*Declaratron*”) is presented in our article “The Mathematical Basis of Semantic Physical Science: Application to Forcefields”.

## Conclusions from the event

There was remarkable and exciting unanimity that semantics should and could be introduced now and rapidly into the practice of large areas of chemistry. We agreed that we should concentrate on the three main areas of crystallography, computation and NMR spectroscopy. In crystallography, this is primarily a strategy of working very closely with the IUCr, being able to translate crystallographic data automatically into semantic form and exploring the value of semantic publication and repositories. The continued development of Chempound for crystal structures is Open and so can be fed back regularly into mainstream crystallography.

The current model of mapping dictionary items onto semantic input/output (rather than more formal ontologies) is generally agreed. We recognised that in some cases, such as parsed logfiles, this has to be done through parsers since the source code or the developer community is not yet able to be FoXified. The ideal situation is for complete semantification of the source code and NWChem will act as a high-profile prototype in QM calculations. We debated the merits of having a single dictionary for computational chemistry as opposed to one dictionary per code and an overarching compchem dictionary. There were proponents of both views and it is therefore necessary for the community to support multiple dictionaries, dictionary migration and “sameAs” functionality. There is clearly value in having a single dictionary for activities such as formal publication, teaching and learning and many other activities.

NMR and other spectroscopy already have prototypic support in the JCAMP dictionaries and legacy parsing software. JCAMP should provide a useful syntactic interoperability but has not been used as widely as we would like. There is also considerable scattered terminology in the printed literature, so that it should be possible to build an NMR dictionary system from existing work.

In all three categories, there is a pressing need for general metadata. This involves concepts such as owner, experiment, environment (data, time, organisation *etc.*) Some of this was prototyped in the oreChem project and has also been codified in projects such as I2S2 [37]. We believe that it is fairly straightforward to create a general dictionary which would support this necessary aspect of physical science semantics.

CML continues to proceed steadily. Martin Dove described it as “a best-kept secret”, *i.e.* it was very valuable but hardly anyone knew of it. This is true of other languages such as content-MathML which has taken over a decade to take root, especially in science. But with a number of scientific markup languages heavily used, and with a wide range of CML implementations we expect it to flourish. And this will catalyse the creation of other similar interoperable approaches across physical science.

## Workshop participants

Peter Murray-Rust

Nico Adams

Sam Adams

Simon Coles

Clyde Davies

Aileen Day

Bert de Jong

Martin Dove

Jorge Estrada

Marcus Hanwell

Brian McMahon

Mahendra Mahey

Karl Mueller

Cameron Neylon

Henry Rzepa

Bill Shelton

Paul Sherwood

Jens Thomas

Jon Tyzack

Andrew Walker

Nancy Washton

Mark Williamson

Erica Yang

## Additional authors at the symposium (not the workshop)

Markus Kraft

Weerapong Phadungsukanan

## Authors' contributions

Both authors have contributed to the development of CML and wrote the manuscript.

## Competing interests

Both authors state that they have no competing interests.
